# Functional connectivity analysis of distracted drivers based on the wavelet phase coherence of functional near-infrared spectroscopy signals

**DOI:** 10.1371/journal.pone.0188329

**Published:** 2017-11-27

**Authors:** Gongcheng Xu, Ming Zhang, Yan Wang, Zhian Liu, Congcong Huo, Zengyong Li, Mengyou Huo

**Affiliations:** 1 Key Laboratory of High Efficiency and Clean Mechanical Manufacture, School of Mechanical Engineering, Shandong University, Jinan, P.R. China; 2 Department of Biomedical Engineering, Faculty of Engineering, The Hong Kong Polytechnic University, Kowloon, Hong Kong, SAR, P.R. China; 3 Beijing Key Laboratory of Rehabilitation Technical Aids for Old-Age Disability, National Research Center for Rehabilitation Technical Aids, Beijing, P. R. China; 4 Key Laboratory of Rehabilitation Aids Technology and System of the Ministry of Civil Affairs, Beijing, P. R. China; National University of Defense Technology College of Mechatronic Engineering and Automation, CHINA

## Abstract

The present study aimed to evaluate the functional connectivity (FC) in relevant cortex areas during simulated driving with distraction based on functional near-infrared spectroscopy (fNIRS) method. Twelve subjects were recruited to perform three types of driving tasks, namely, straight driving, straight driving with secondary auditory task, and straight driving with secondary visual vigilance task, on a driving simulator. The wavelet amplitude (WA) and wavelet phase coherence (WPCO) of the fNIRS signals were calculated in six frequency intervals: I, 0.6–2 Hz; II, 0.145–0.6 Hz; III, 0.052–0.145 Hz; IV, 0.021–0.052 Hz; and V, 0.0095–0.021 Hz, VI, 0.005–0.0095Hz. Results showed that secondary tasks during driving led to worse driving performance, brain activity changes, and dynamic configuration of the connectivity. The significantly lower WA value in the right motor cortex in interval IV, and higher WPCO values in intervals II, V, and VI were found with additional auditory task. Significant standard deviation of speed and lower WA values in the left prefrontal cortex and right prefrontal cortex in interval VI, and lower WPCO values in intervals I, IV, V, and VI were found under the additional visual vigilance task. The results suggest that the changed FC levels in intervals IV, V, and VI were more likely to reflect the driver’s distraction condition. The present study provides new insights into the relationship between distracted driving behavior and brain activity. The method may be used for the evaluation of drivers’ attention level.

## Introduction

Car driving has become an inevitable social activity and requires higher brain functions even for basic driving operations and driving safety [1]. Safe car driving is highly important. No other system exposes many individuals to as much high risk, especially risk to their lives, as that in a car [2]. According to statistics, 25%-50% of road traffic accidents involved distracted drivers, whereas 36.4% involved those who participate in in-vehicle secondary task (IVST) behavior [3]. IVST refers to other tasks indirectly related or unrelated to driving, such as listening to radio, looking at maps, talking with passengers, or adjusting car facilities. These tasks occupy the driver’s visual, cognitive, and motion resources and distract the driver’s attention to varying degrees [4,5]. Many studies have proven that IVST seriously affects a driver’s driving performance and traffic safety [6–8]. Driving a vehicle safely requires concentration, divided attention to visual and auditory events, and cognitive decision making in a variable environment [9]. Drivers’ mental load and distraction level increase with the introduction of new interfaces and technologies in vehicles. Therefore, evaluating the attention status and brain activity of drivers when studying distracted driving is necessary.

Functional connectivity (FC) is defined as a strong temporal correlation between two raw time series in a low-frequency band, and cardiopulmonary interval signal frequencies are not included [10]. FC reflects some essential connections in cerebral cortex, and this functional network reveals an intrinsic structural brain network [11,12]. FC has been broadly used in various driving studies to evaluate the correlation levels in drivers [1,13–15]. Studies show that distracted driving greatly affects a driver’s brain activity. The simultaneous understanding task of the language made the mental resources far away from driving [16]. The distracted drivers’ brain sacrificed important visual attention and alertness functions from the posterior cortex to recruit sufficient brain resources to perform secondary cognitive tasks [9]. However, little information on the effects of distraction on brain FC in distracted driving is known.

Studies on FC based on functional near-infrared spectroscopy (fNIRS) were continuously conducted in recent years. fNIRS is a novel, versatile, nondestructive blood oxygen testing technique widely used in brain function research and neuronal imaging [17–20]. fNIRS indirectly detects neuronal brain activity by recognizing changes in oxygenated and deoxygenated hemoglobin concentrations (Delta [HbO_2_] and Delta [dHb], respectively) in an area of interest in the cortex; moreover, fNIRS is applied in studies on brain computer interfaces [21–25]. fNIRS offers unique advantages, such as portability, convenience, and a combination of advisable temporal and spatial resolutions, compared with other brain imaging techniques, such as functional magnetic resonance imaging or electroencephalography [26–28].

Blood oxygen signals detected by fNIRS possess a strong time-frequency characteristics and involve different physiological sources in various frequency intervals [29,30]. Fluctuant fNIRS signals mainly involve neurovascular coupling and systemic physiological processes. Physiological processes, such as respiration and evoked or non-evoked neurovascular coupling that cause hemodynamic changes in fNIRS signal, do not occur as separate and non-influenced processes but rather as interrelated processes [20]. Wavelet-based method has been used to identify different characteristic frequencies of cerebral oxygenation signals [31,32] and distinguish main physiological noise contributions by combining concurrent time-domain fNIRS and peripheral physiological recordings [33]. Wavelet phase coherence (WPCO) can be used to assesses the correlation between two signals with their phase information and evaluates the possible connectivity based on the degree of coincidence of the instantaneous phase over the entire time series [34].

The prefrontal cortex (PFC) has been described in numerous occasions as a cognition cortical area in humans (e.g., [35,36]). PFC is an appropriate research object in investigating attention-related changes in brain activity. The motor cortex (MC) is important in sensation and motor control [37]. The occipital cortex (OC) mainly functions in processing of visual information, and the primary visual cortex V1 is located in OC [38]. Studies have demonstrated that the prefrontal, motor, and occipital cortices as brain regions responsible for various driving maneuvers [9,39,40]. Hence, we hypothesized that PFC, MC, and OC are involved in distracted driving involving visual and auditory tasks. This study measures fNIRS signals in these regions of interest during distracted driving and assesses the change in FC by performing WPCO analysis of the fNIRS signals.

## Materials and methods

### Subjects

A total of 12 subjects (5 females and 7 males) were recruited from Shandong University to participate in this study. All of the subjects had driving license but the driving experiment was less than one year. We excluded subjects with the following characteristics: history of hypertension, neurological or psychiatric disease, and drinking or smoking habit. Hypertension was defined as systolic blood pressure ≥ 140 mm Hg or diastolic blood pressure ≥ 90 mm Hg [41]. The experimental methods were approved by the Shandong University Human Ethics Committee and carried out according to the ethical standards of the 1964 Helsinki Declaration. All the participants provided written informed consent before participating in the study.

Table 1 shows the age of the subjects, body mass index (BMI), and blood pressure measured before the experiment.

**Table 1:** Basic information of the subjects.

Parameters	Means (Standard deviations)
Age (years)	24.4 (1.2)
Body mass index (BMI)	21.3 (2.9)
Systolic blood pressure (mmHg)	115.7 (4.8)
Diastolic blood pressure (mmHg)	69.7 (4.5)

Values are presented as means with standard deviations.

### Procedures

**Fig 1:**
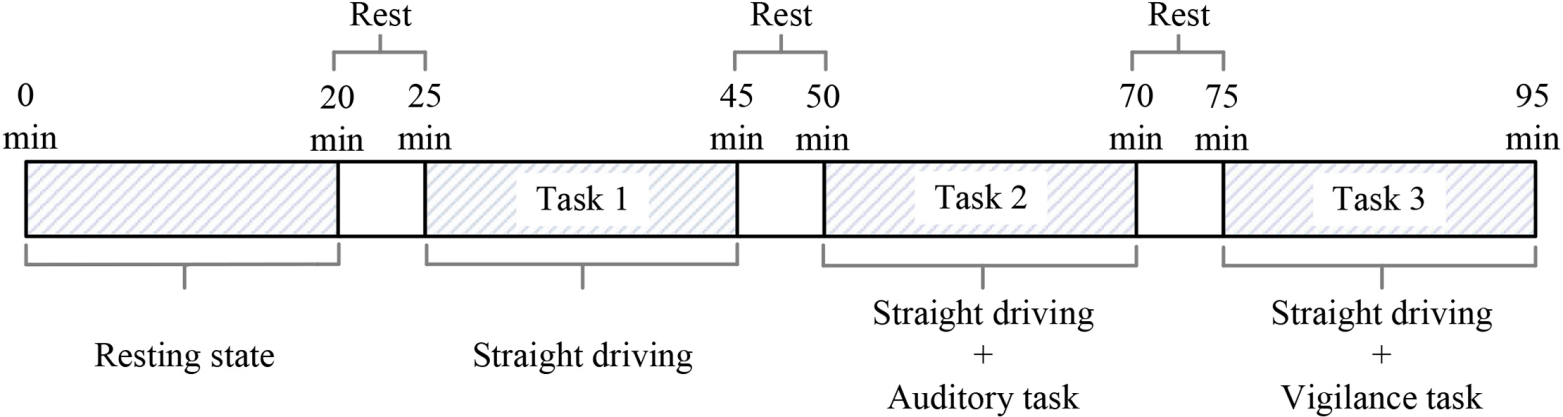
Experimental procedures.

A novel driving simulator combined with a semi-immersive virtual reality technology, throttle and brake pedals, and a steering wheel was used to simulate the driving environment in this study. The experiment was divided into four states, namely, resting state, straight driving (Task 1), straight driving with an audio task (Task 2), and straight driving with a visual vigilance task (Task 3). Each state continued for 20 min, and there were 5 minutes between each state in order to avoid the impact of driving fatigue. Fig 1 shows the experimental procedures.

**Fig 2:**
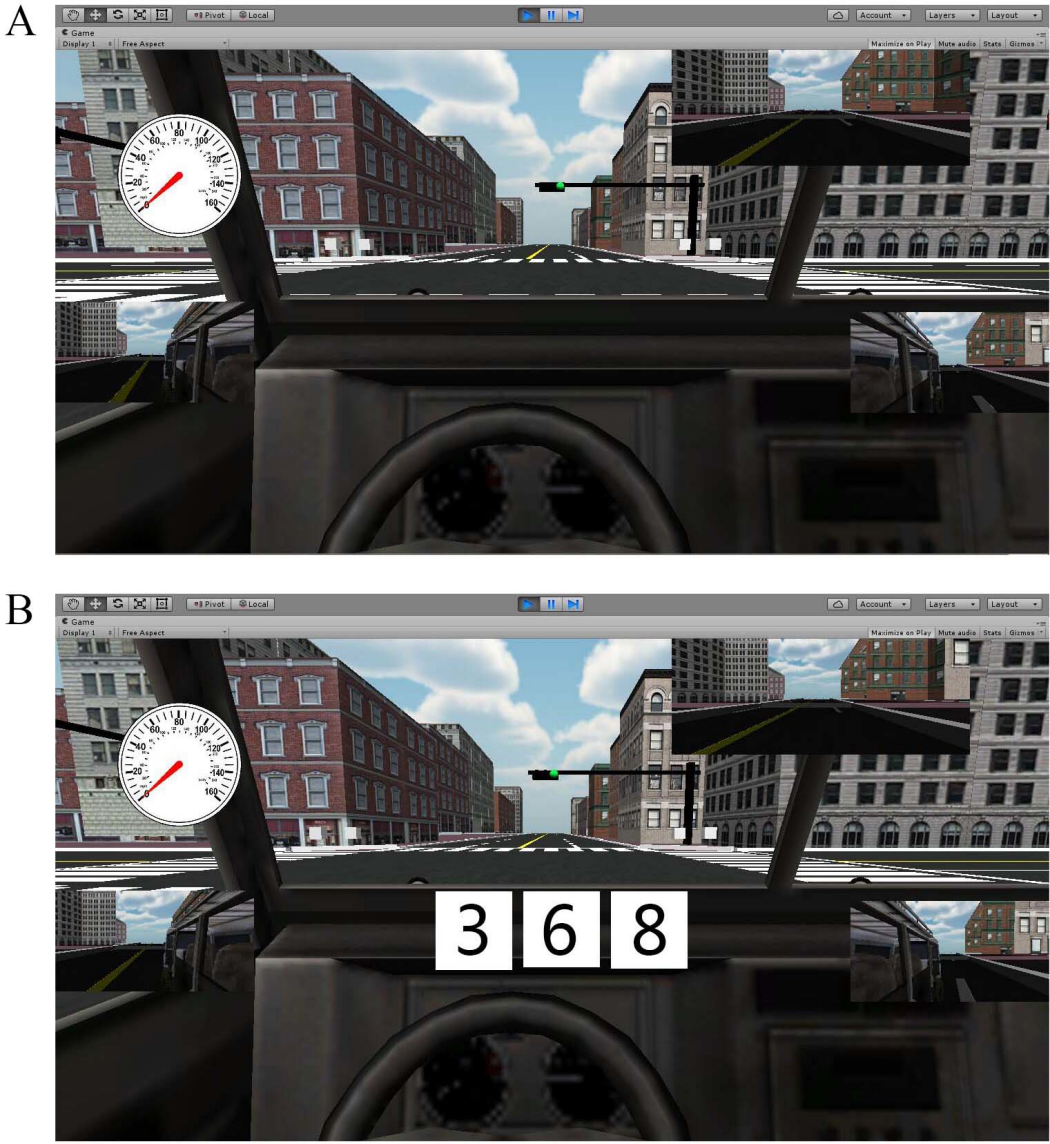
Driving scenarios in the experiment. Fig A shows the scenario applied to straight driving and straight driving with auditory task. Fig B shows the scenario applied to straight driving with vigilance task.

During resting state, subjects were required to sit comfortably in the driving simulator, eyes fixed at the scene on screen (Fig 2A), hands on the steering wheel, and foot on the brake pedal. Straight driving involves subjects driving straight forward along the virtual road in the driving simulator. The subjects are in a focused condition in this state. The auditory task was designed to simulate the distraction caused by ambient sound during driving. Subjects were asked by the experimenter to mentally solve two-digit addition and subtraction problems and relay the answer. The vigilance task was described in a previous study [42]. This task was applied in our experiment to simulate distraction from image or visual change. Fig 2B shows the arrangement of vigilance tasks in Task 3. Subjects were required to depress the brake pedal upon the appearance of three different odd numbers. Before the experiment, subjects were given 10 min to adjust to these states. fNIRS measurement was implemented continuously throughout the experiment.

### Measurement

**Fig 3:**
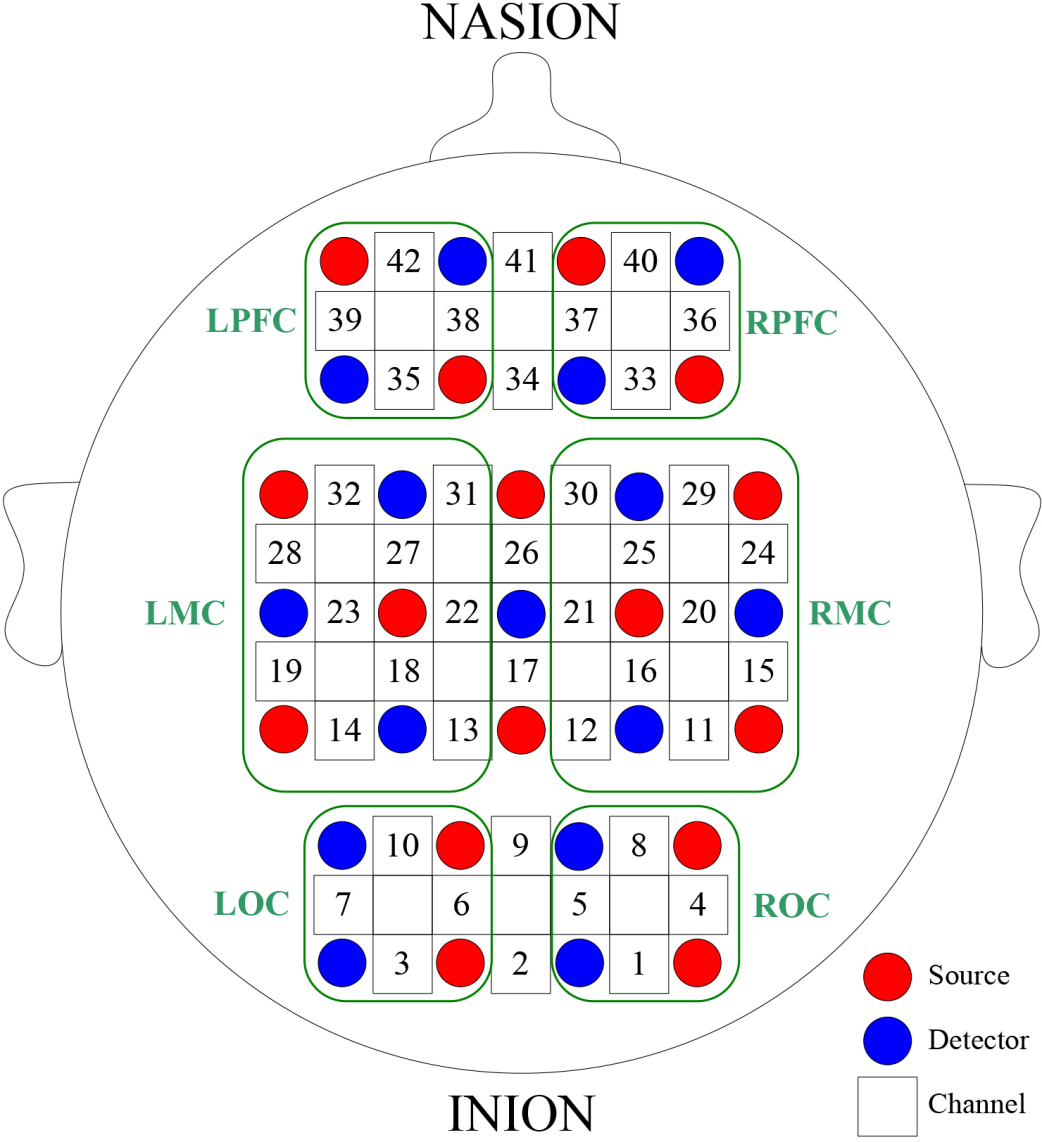
Configuration of source optodes (red dots), detector optodes (blue dots), and measurement channels (black numbers). Six cerebral cortex areas are separate by the green frames as LPFC, RPFC, LMC, RMC, LOC and ROC.

A multi-channel tissue oxygenation monitor (NirScan, Danyang Huichuang Medical Equipment Co., Ltd) was used in NIRS measurements. The calibration function of the instrument and corresponding template were used to ascertain the channels to fall exactly in correspondence of the 10/10 electrode positions with the different head size of the participants. The template was placed at the corresponding positions of PFC, MC, and OC. The template and the head were fixed with an elastic band. When placing the probes into the template, the subjects’ hair was pulled aside to ensure that the probes were in direct contact with the scalp. Optodes and templates were placed on the following cortical regions: left prefrontal cortex (LPFC), right prefrontal cortex (RPFC), left motor cortex (LMC), right motor cortex (RMC), left occipital cortex (LOC), and right occipital cortex (ROC), and the inter-optodes distance was 30 mm. A total of 42 channels corresponding to the 10/10 system were formed [43]. Fig 3 shows the configuration of the source optodes, detector optodes, and measurement channels. The 36 channels enclosed by the green box (LPFC, RPFC, LMC, RMC, LOC and ROC) in Fig 3 were included in the analyses. Signals with a low signal-to-noise ratio were removed, and the sampling rate was 10 Hz.

Subjects were required to refrain from moving or shaking their heads during the experiment. In the experimental process of resting state, the template of one subject fell off, so only 11 subjects’ data are valid in the state. The driving performance parameters, including driving speed and driving errors (DE) (times of leaving the driveway and inappropriate crossing of lines) were recorded by the simulator.

http://dx.doi.org/10.17504/protocols.io.kcjcsun

### Data pre-processing and wavelet-based coherence analysis

**Fig 4:**
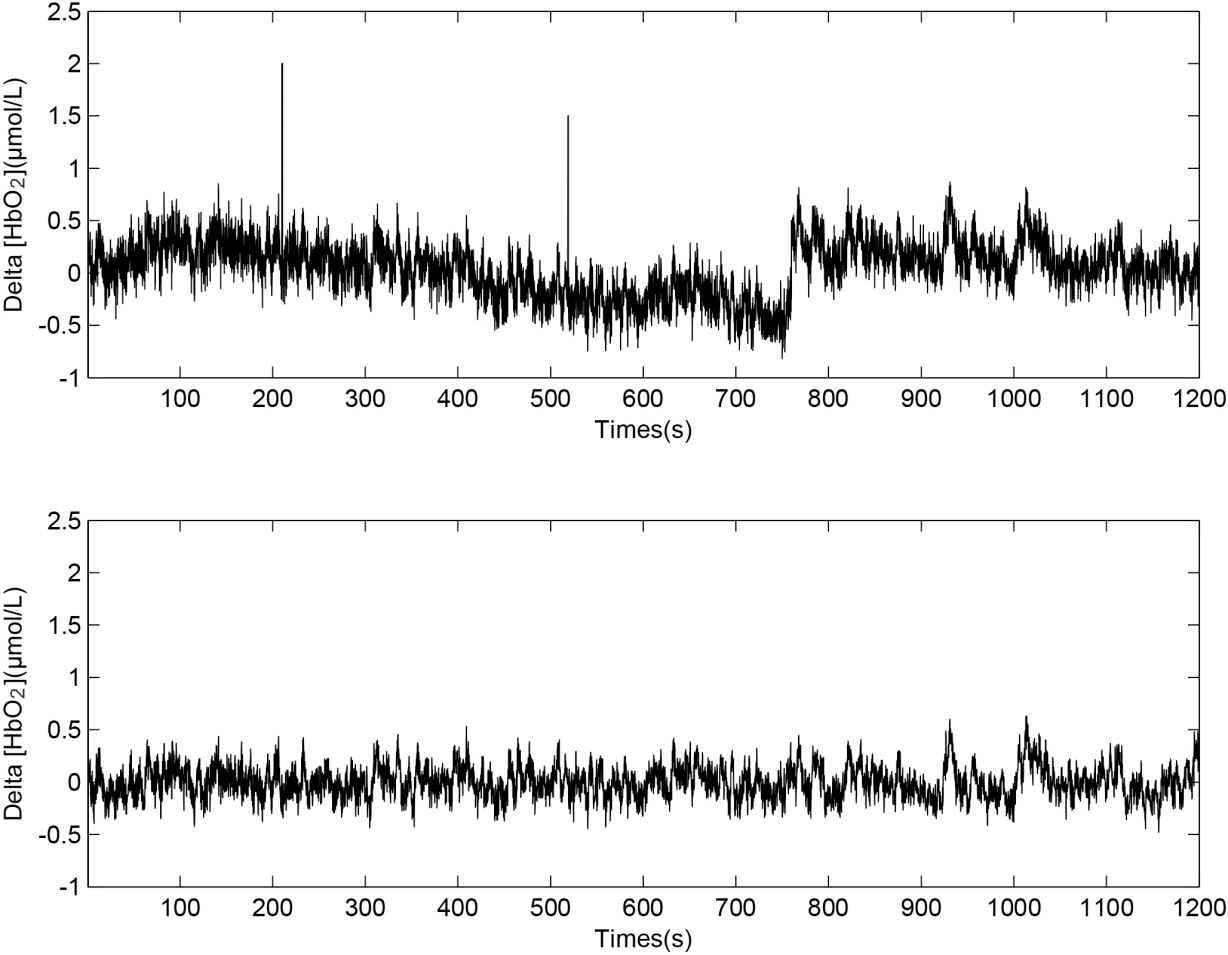
Delta [HbO2] signals before (A) and after (B) data preprocessing.

The pre-processing method for fNIRS data has been described in detail in previous studies [44,45]. In short, we used the moving average method and a six-order Butterworth band-pass filter to obtain the filtered signals of 0.005–2 Hz with an improved signal-to-noise ratio. The time window used for the moving average filter was 3s. Movement artifacts appearing on visually identified channels were removed by using moving standard deviation and spline interpolation routines [46]. Fig 4 shows the original Delta [HbO_2_] signal (Fig 4A) and the preprocessed Delta [HbO_2_] signal (Fig 4B). The standard deviation of speed (SDS) was calculated from the driving speed to reflect the degree of dispersion in each state.

WPCO analysis was described in our previous studies [47–50]. In brief, wavelet transform (WT) is a method facilitating complex transformations of time series from the time domain to the time-frequency domain and provides appropriate time and frequency resolutions by using tunable filter band lengths. In this study, Morlet wavelet was used for continuous WT as it has its best time-frequency compactness [51]. This compactness is obtained by measuring the area of the time-frequency box corresponding to different wavelet. Morlet wavelet shows the most likely small time-frequency box area in all wavelet functions, and thus can achieve the best time-frequency resolution.

Through WT, we can obtain the wavelet amplitude (WA) of each Delta [HbO_2_] signals. WA is the average result of the WT in the time domain. By subtracting the instantaneous phase, we can obtain the instantaneous phase difference of two Delta [HbO_2_] signals: Δ*ϕ*(*f*,*t*_*n*_). We subsequently averaged the cos Δ*ϕ*(*f*,*t*_*n*_) and sin Δ*ϕ*(*f*,*t*_*n*_) in the time domain to obtain 〈cos Δ*ϕ*(*f*,*t*_*n*_)〉 and 〈sin Δ*ϕ*(*f*,*t*_*n*_)〉. Furthermore, WPCO was defined as:

$$C_{\phi }(f)=\sqrt{{\langle \cos\;\Delta \phi (f,t_{n})\rangle }^{2}+{\langle \sin\;\Delta \phi (f,t_{n})\rangle }^{2}}$$ (1)

WA reflects the magnitude of the fluctuation of the original signal at a certain frequency, so it serves as an index of power that can be used to describe the activity intensity of the cortical region. The typical hemodynamic response to mental activation is the basis for NIRS measurement. When a specific brain area is activated, neural metabolism is supported through a localized vascular response that causes an influx of oxygen-rich blood to the active area and the surrounding tissue. This phenomenon leads to an increase in [HbO_2_] and a decrease in [dHb] in the active brain area [52,53]. The functional hyperaemia mechanism is to adjust the distribution of cerebral blood flow (CBF) based on functional activities of different brain regions [54]. Therefore, when the activity of the cerebral cortex region increases or decreases, the flow into this area changes accordingly. This change is reflected in the WA of the fNIRS signal ([HbO_2_]) during driving task, which is used to characterize the intensity of activity in a particular cortex region. In this study, brain activation represents the task-evoked cortical activity related to the execution of the task.

WPCO identifies possible relationships by evaluating the match between the instantaneous phases of two signals. Cerebral NIRS signals mainly consist of evoked neurovascular coupling, non-evoked neurovascular coupling, and systemic physiological interference [20]. Some different, and possibly overlapping, physiological mechanisms such as the sympathetic nervous system, endothelial derived nitric oxide, and vascular myogenic responses could play some part in neurovascular coupling [55]. We previously demonstrated the oscillations in NIRS signals with six characteristic frequencies [49,56,57], possibly reflecting neurovascular coupling and systemic regulation activities. Overall, six frequency intervals were divided in WA and WPCO analyses based on their possible physiological origins: I, 0.6–2 Hz (cardiac activity); II, 0.145–0.6 Hz (respiration); III, 0.052–0.145 Hz (myogenic activity); IV, 0.021–0.052 Hz (neurogenic activity); and V, 0.0095–0.021 Hz (NO-related endothelial metabolic activity), VI, 0.005–0.0095Hz (NO-independent endothelial activity) [58].

Spurious correlations (false discoveries) across two channels of data may be produced by self-correlations of fNIRS signals for WPCO analysis. These self-correlations are closely related to error terms, such as slow hemodynamic signal, systemic physiological noise, and motion artifacts, which may lead to either high false-discovery rates or high false-negative rates in correlation analyses [59]. Low-frequency components are fewer than high-frequency components in finite length signals. Less variation in phase difference occurs at low frequencies due to error terms and results in increased phase coherence. The amplitude-adaptive Fourier transform (AAFT) method was applied to perform significant WPCO test. A total of 100 surrogate signals were produced, and these signals possess the same mean, variance, and autocorrelation functions as the original signal but without any phase correlation. The WPCO value of the experimental signal was considered significant when it was higher than the mean WPCO values of surrogate with two standard deviations. This significant value denotes significant connectivity [34].

The WA and WPCO values in the specific frequency interval were obtained by using the averaging method. In one frequency interval, six WA values of each subject were obtained by averaging the WA of the internal channels in each brain cortex (LPFC, RPFC, LMC, RMC, LOC and ROC). The 15 WPCO values were obtained from each subject by averaging the WPCO of channels between two brain regions (LPFC-RPFC, LPFC-LMC, LPFC-RMC, LPFC-LOC, LPFC-ROC, RPFC-LMC, RPFC-RMC, RPFC-LOC, RPFC-ROC, LMC-RMC, LMC-LOC, LMC-ROC, RMC-LOC, RMC-ROC and LOC-ROC). This process was performed in six frequency intervals.

In this study, the connectivity of six frequency intervals was classified into global connectivity (GC) and FC. FC is usually characterized by a temporal correlation between two raw time series with low frequency (<0.1 Hz) [19] and separable from respiratory (0.1–0.5 Hz) and cardiovascular (0.6–1.2 Hz) signal frequencies [10]. The systemic signals including respiratory and cardiovascular signals were commonly considered global interferences [60]. In this study, FC was revealed by the significant WPCO values in intervals III to VI (0.005–0.145 Hz), whereas GC was revealed by the significant WPCO values in intervals I to II (0.145–2 Hz). These parameters might reflect the neurovascular coupling and systemic regulation activities. The GC reflects the synchronization of cardiac and respiratory activities in the cerebral areas [61].

### Statistical analysis

The normal test (Kolmogorov-Smirnov test) and variance uniformity test (Levene test) of each subject’s data were performed at the group level to ensure that the assumptions required for parameter analysis were satisfied. Significant differences in the driving performance parameters and WA, as well as WPCO changes in each state, were calculated through one-way ANOVA. A difference of p<0.05 was considered statistically significant. The Bonferroni t-test was used for the pair-wise comparisons. Three groups for WA and WPCO comparison were designed (rest and Task 1, Task 1 and Task 2, Task 1 and Task 3), so the α value was set to 0.0167 (0.05/3). Two groups for driving performance comparison were designed (Task 1 and Task 2, Task 1 and Task 3), thus the α value was set to 0.025 (0.05/2). Pearson correlation coefficient test was performed to compare the changes between WPCO (△WPCO) and driving performance parameters (△SDS and △DE) under the influence of different secondary tasks. Δ represents the difference between task 1 and task 2, or between task 1 and task 3.

## Results

### Driving performance parameters

**Fig 5:**
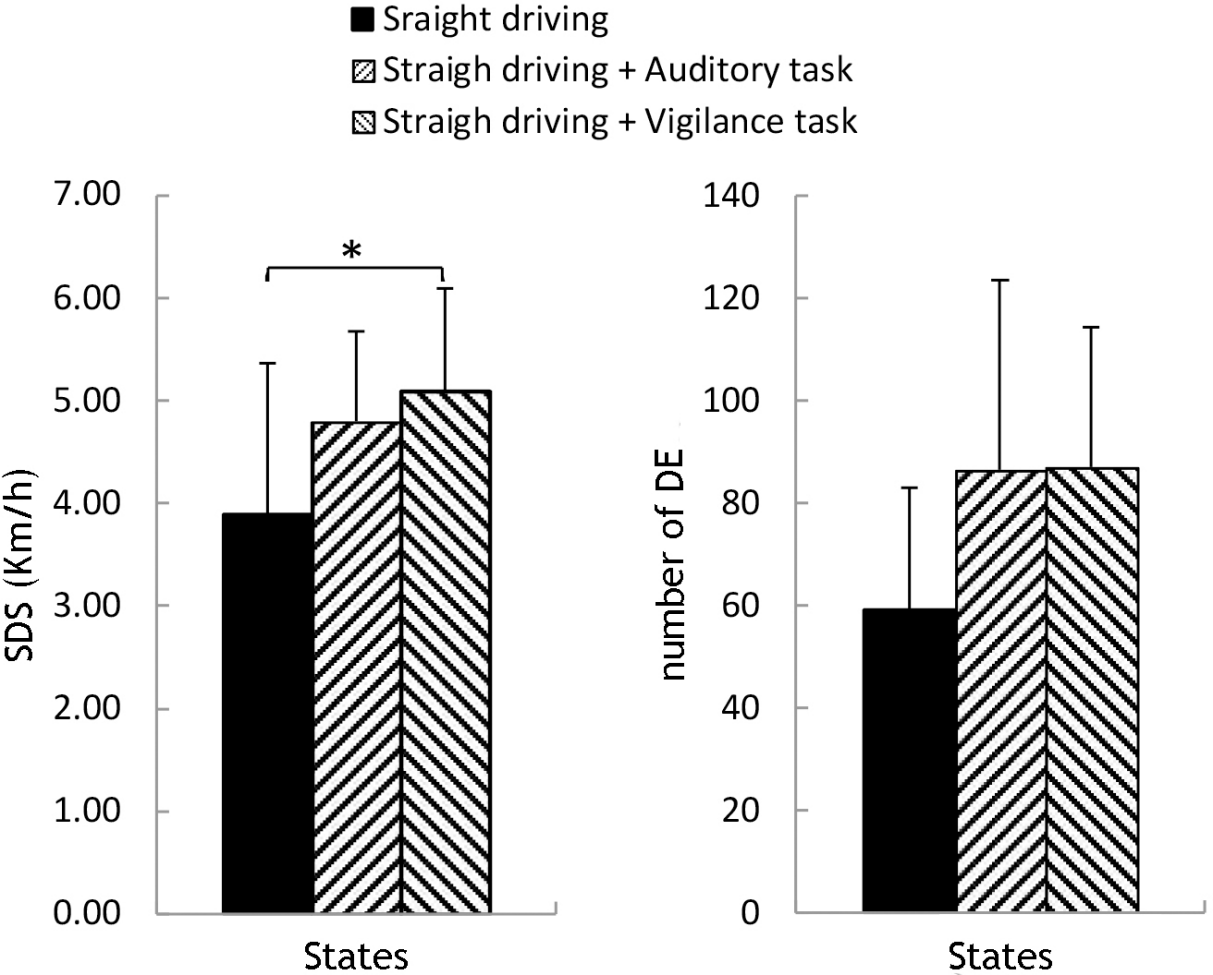
Comparison of SDS and DE between Task 2 and Task 1, Task 3 and Task 1. Significant differences are marked with *(P (t-test) <0.025).

Fig 5 illustrates the changes in driving performances between straight driving and the other two distracted states. The SDS in Task 3 was significantly higher than that in Task 1 (F (2, 33) = 3.564, p = 0.040, p (t-test) = 0.015).

Table 2 shows a list of the Pearson correlation results. Significant negative correlation existed between △SDS and △WPCO in LPFC-RMC (interval III) between Task 1 and Task 2 (-0.8< R < -0.5, p < 0.05). Similar negative correlation was observed in LPFC-RPFC (interval I and IV) and RPFC-ROC (interval IV) between Task 1 and Task 3. Furthermore, significant positive correlation existed between △SDS and △WPCO in RPFC-LMC (interval V), LMC-LOC (interval I) and LOC-ROC (interval VI) (0.5< R < 0.8, p < 0.05) between Task 1 and Task 2.

**Table 2:** Correlations between ΔWPCO and ΔSDS, ΔWPCO and ΔDE.

	Task	Cortex areas	Intervals	ΔSDS (Km/h)		ΔDE	
				R	P	R	P
**ΔWPCO**	Task 1 and Task 2	LPFC-RMC	III	-0.610	0.035*	-0.405	0.192
		RPFC-LMC	V	0.534	0.074	0.586	0.045*
		LMC-LOC	I	0.401	0.197	0.625	0.030*
		LOC-ROC	VI	0.398	0.201	0.653	0.021*
	Task 2 and Task 3	LPFC-RPFC	I	-0.579	0.048*	-0.107	0.740
			IV	-0.618	0.032*	0.095	0.769
		RPFC-ROC	IV	-0.585	0.046*	-0.105	0.746

Δ represents the difference between Task 1 and Task 2, or between Task 1 and Task 3.

Significant correlations are marked with

*(p < 0.05).

### Connectivity maps

**Fig 6:**
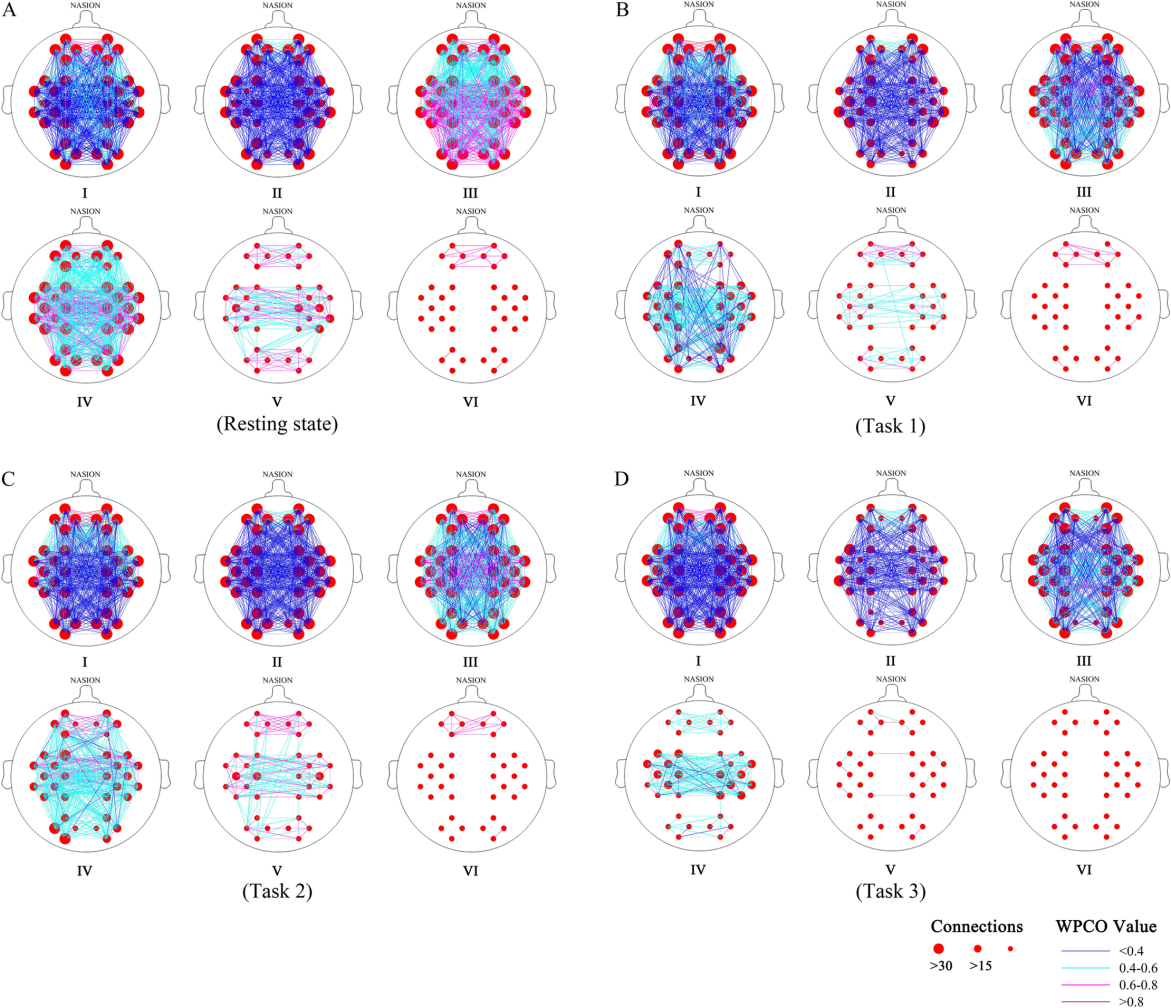
**FC (frequency intervals III to VI) and GC (frequency intervals I and II) maps of resting state (A), Task 1 (straight driving) (B), Task 2 (straight driving with auditory task) (C) and Task 3 (straight driving with vigilance task) (D).** The connectivity line indicated a significant WPCO value between two channels. The color of line indicates the connectivity intensity, and the sizes of the red dots indicate the numbers of connectivity among the channels.

Fig 6 shows the connectivity maps in the six frequency intervals under the four states at the group level. The GC maps were formed by the significant WPCO in intervals I and II, while the FC maps were formed by those in intervals III to VI. The connectivity line indicated a significant WPCO value between two channels. The color of line indicates the connectivity intensity, and the sizes of the red dots indicate the numbers of connectivity among the channels.

The comparison of connectivity was performed between Task 1 and resting state, Task 1 and Task 2, Task 1 and Task 3. The connectivity was dense in intervals I to IV, and sparse in intervals V and VI in each state. The connectivity map was sparser and the connectivity was weaker in intervals II to V in straight driving than in the resting state (Fig 6(B) and Fig 6(A)). The connection map showed evident increase in interval II in Task 2 compared with Task 1. Also, the connection map showed evident increase in interval IV between RPFC and other cortex areas, in interval V between PFC and LOC, LMC and LOC, LMC and RMC in Task 2 compared with Task 1 (Fig 6(C) and Fig 6(B)). However, the map showed a decrease in all the intervals, especially in intervals IV to VI in Task 3 compared with that in Task 1 (Fig 6(D) and Fig 6(C)).

### Connectivity matrices

**Fig 7:**
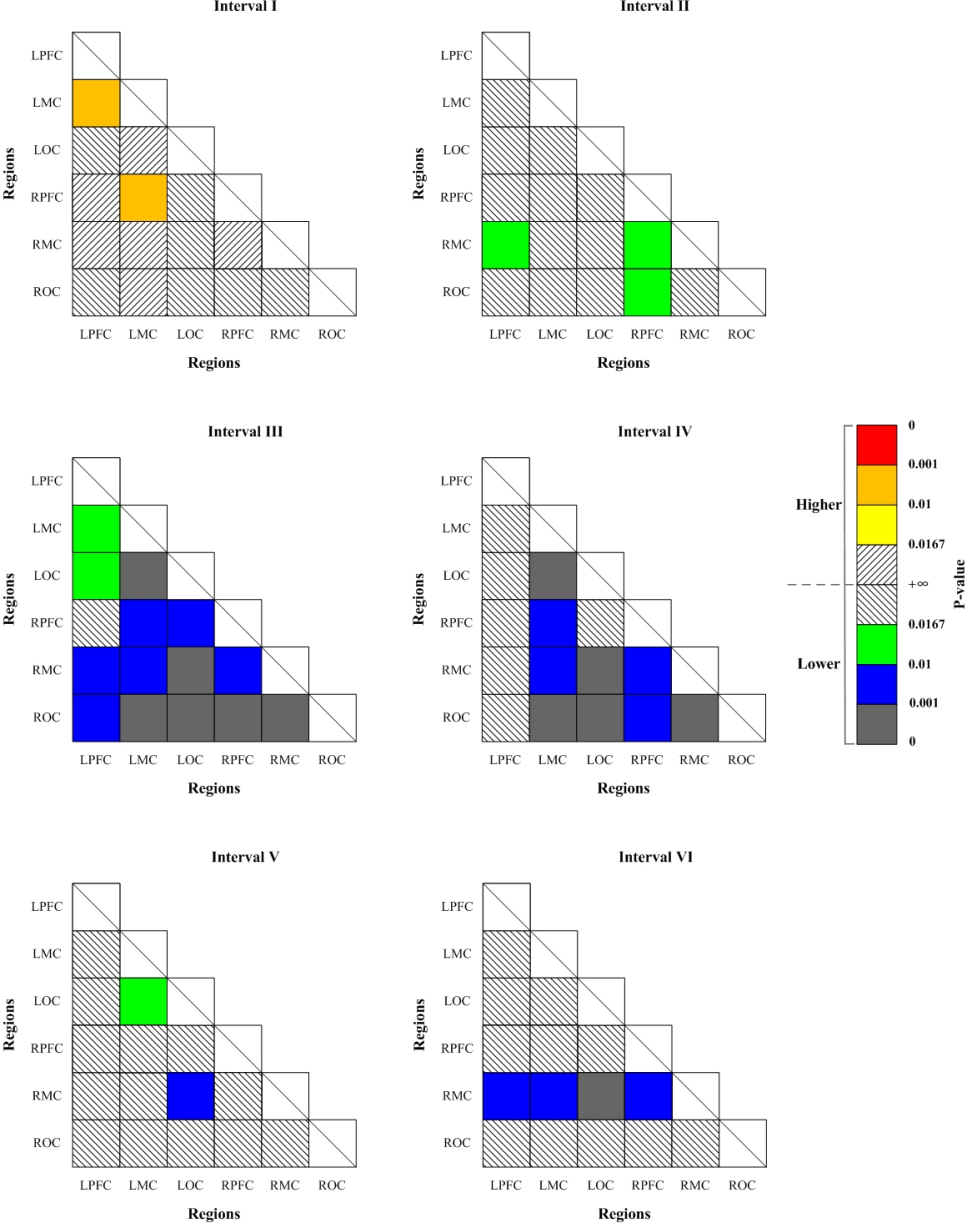
Significance matrices representing significantly changed WPCO value in comparison with Task 1 to resting state (indicated by the p-value of Bonferroni t-test). Warm colors represent increase of WPCO value, brighter boxes represent stronger significance. Cold colors represent decrease of WPCO value, darker boxes represent stronger significance.

Fig 7 shows the significant changes (indicated by the p-value of Bonferroni t-test) of WPCO values as revealed by the one-way ANOVA analysis between Task 1 and resting state. The WPCO values of LPFC-LMC and LMC-RPFC were significantly higher in interval I in straight driving state compared with the resting state. On the contrary, significantly lower WPCO values were found in interval II between LPFC and RMC, RPFC and ROC, RPFC and RMC, in interval III in all cortex regions except for LPFC-RPFC, in interval IV in all cortex regions except for LPFC-ROC, LPFC-RMC, LPFC-RPFC, LPFC-LOC, LPFC-LMC and RPFC-LOC, in interval V between LMC and LOC, LOC and RMC, in interval VI between RMC and LPFC, RMC and LMC, RMC and LOC, RMC and RPFC, in straight driving state compared with the resting state.

**Fig 8:**
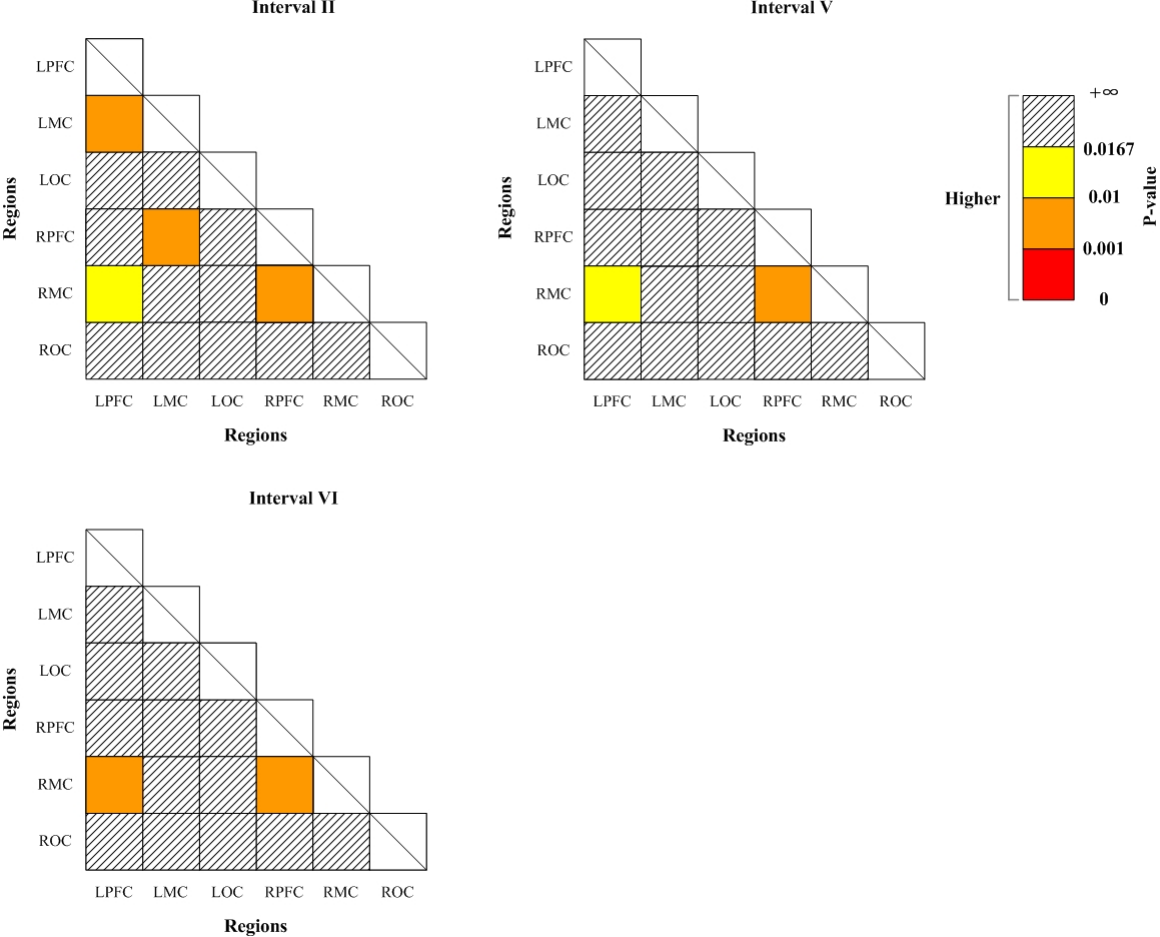
Significance matrices representing significantly changed WPCO value in comparison with Task 2 to Task 1 (indicated by the p-value of Bonferroni t-test). Warm colors represent increase of WPCO value, brighter boxes represent stronger significance.

Fig 8 shows the significant changes (indicated by the p-value of Bonferroni t-test) of WPCO values between Task 2 and Task 1. Significantly higher WPCO values were found in intervals II, V and VI in Task 2 compared with the values in Task 1 (II: LPFC-RMC, LPFC-LMC, LMC-RPFC, and RPFC-RMC; V: LPFC-RMC and RPFC-RMC; VI: LPFC-RMC and RPFC-RMC).

**Fig 9:**
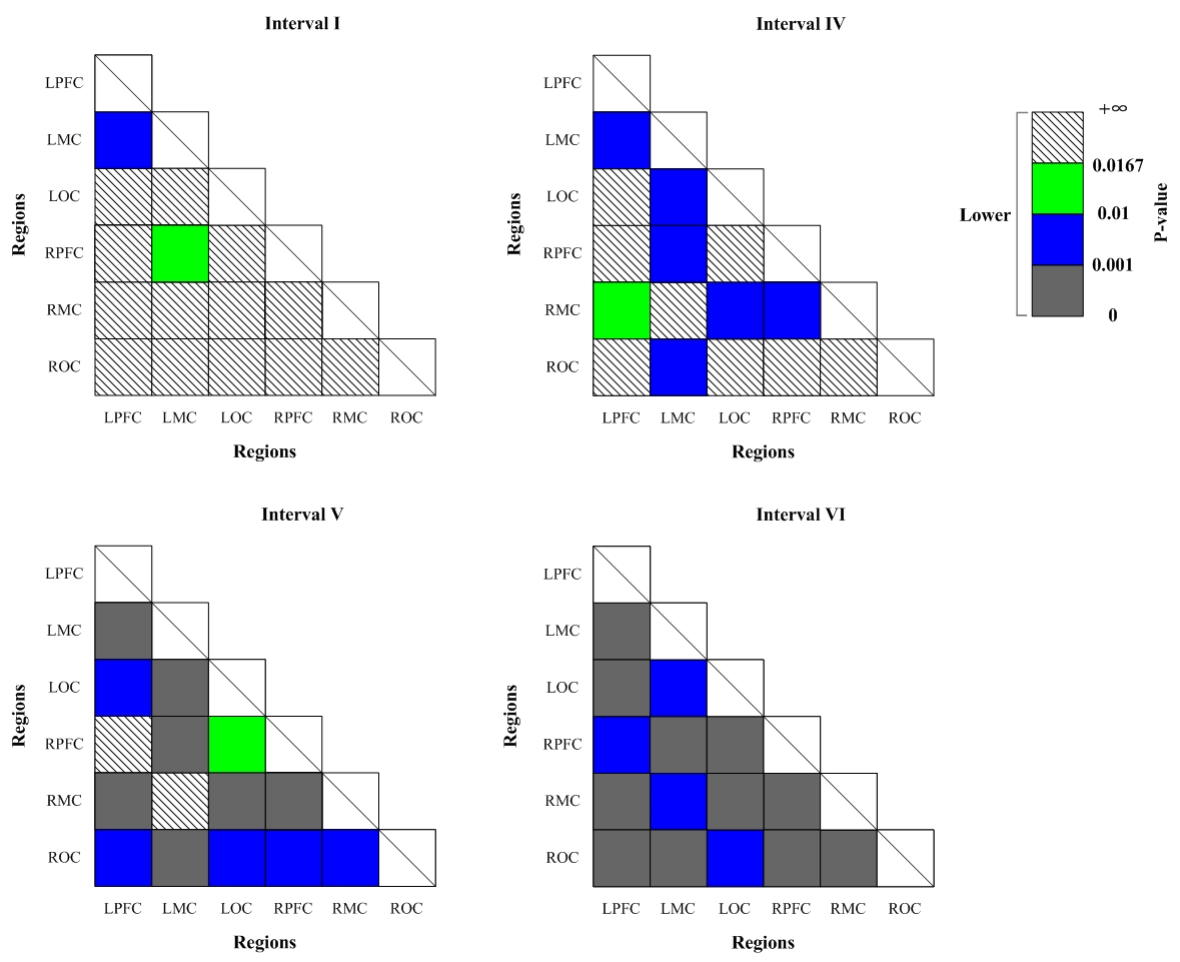
Significance matrices representing significantly changed WPCO value in comparison with Task 3 to Task 1 (indicated by the p-value of Bonferroni t-test). Cold colors represent decrease of WPCO value, darker boxes represent stronger significance.

Fig 9 shows the significant changes (indicated by the p-value of Bonferroni t-test) of WPCO values between Task 3 and Task 1. Significantly lower WPCO values were found in intervals I between LPFC and LMC, LMC and RPFC, in interval IV in LPFC-RMC, LPFC-LMC, LMC-ROC, LMC-RPFC, LMC-LOC, LOC-RMC and RPFC-RMC, in interval V among all cortex regions except for LPFC–RPFC and LMC-RMC, in interval VI among all cortex regions in Task 3 compared with Task 1.

### WA values

**Fig 10:**
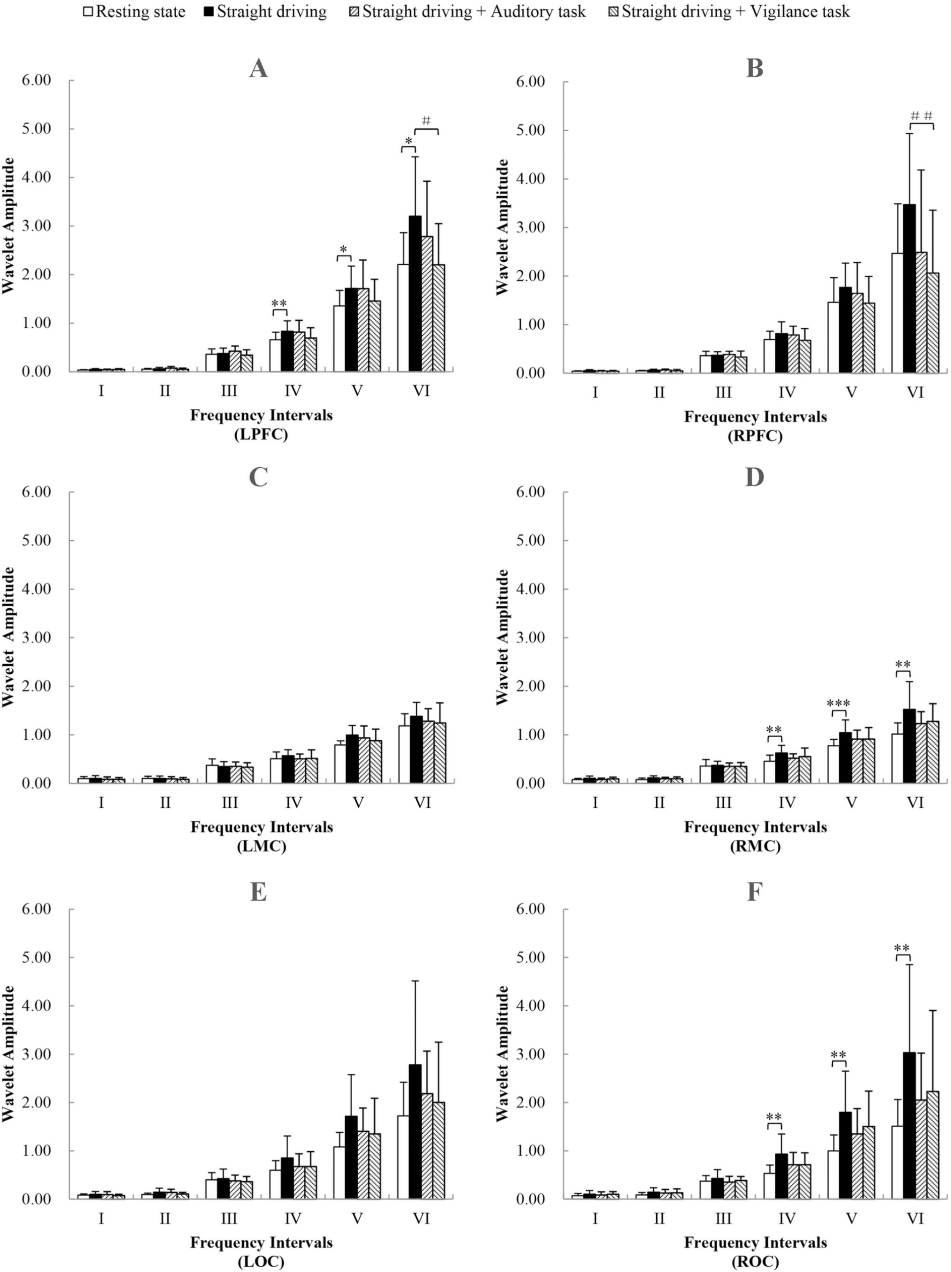
Comparison of the WA value in the six frequency intervals in the LPFC (A), in the RPFC (B), in the LMC (C), in the RMC (D), in the LOC (E) and in the ROC (F). Significant differences between resting state and Task 1 are marked with * (P (t-test) <0.0167), ** (P (t-test) <0.01), *** (P (t-test) <0.001). Significant differences between Task 1 and Task 3 are marked with # (P (t-test) <0.0167), ## (P (t-test) <0.01).

Fig 10 compares the WA values of the six frequency intervals in each cortex area. The comparison of WA values was performed between Task 1 and resting state, Task 1 and Task 2, Task 1 and Task 3. Significantly higher WA values in intervals IV, V, and VI were found in the LPFC, RMC and ROC in straight driving sate compared with the resting state. Moreover, significantly lower WA values in interval VI were found in the LPFC (F (3, 43) = 3.333, p = 0.028, p (t-test) = 0.010) and RPFC (F (3, 43) = 2.853, p = 0.048, p (t-test) = 0.007) in Task 3 than in Task 1.

## Discussion

In the present study, higher brain activation in intervals IV to VI and decreased connectivity in intervals II to VI were found in straight driving state than that in resting state. Also, the WPCO in intervals II, V, and VI showed enhanced level as a result of additional auditory task, but decreased level in intervals I, IV, V, and VI due to additional visual vigilance tasks. Moreover, driving performance became worse and brain activation decreased when secondary tasks were applied to simulated driving.

The analysis of connectivity in fNIRS is based on the statistical relationship between spontaneous temporal fluctuations between two or more parts of the brain. Such analysis may be performed in the time domain or in the frequency domain using spectral coherence or phase locking measurements [59]. In this study, FC or GC was identified with phase synchronization. The phase information was extracted by multiple scales WT, and the WPCO method was used to identify oscillations with consistent phase differences. Consistent phase differences at a certain frequency interval indicated high phase synchronization.

### GC and FC in straight driving state and the resting state

This study showed a significantly lower level of GC in straight driving than in the resting state in the LPFC-RMC, RPFC-ROC and RPFC-RMC in interval II. The oscillations in interval II closely resemble the oscillations of respiratory activity [62]. Driving action requires energy provided by strong respiratory activity, which produce significant changes in the CBF independent of task-related nerve activation [63]. The increase of this task-related CBF was revealed by the rising WA values in straight driving. However, the activation levels of cortex regions were different under the driving task and the oxygen extraction fractions might vary from one region to another [64]. The declined GC in interval II in some cortex regions may be attributed to the inhomogeneous activation.

The oscillations in frequency interval III correspond to the myogenic activity of smooth vascular muscle cells [58,61]. Vascular smooth muscle contracts in response to increased intravascular pressure. This contraction is then relieved by reduced pressure and directly regulates the diameter and pressure of blood vessels. In the multi-cognitive task, such as driving, human manifestations are immediately affected by undifferentiated resources [65]. Thus, the vascular smooth muscles contract in response to the incremental intravascular pressure, and the amplitude of myasthenia is increased [58]. That is, the myogenic mechanism affects FC levels. Comparing straight driving to resting state, the raising WA values in interval III in brain region indicate the difference of myogenic activity, especially between LMC and other regions. Correspondingly, the reduced synchronization of the vascular smooth muscles’ spontaneous contraction was revealed by the significantly lower WPCO values in all connectivity except for LPFC-RPFC, which indicates lower level of FC in interval III.

The oscillations in frequency interval IV correspond to the neurogenic activity controlling smooth muscle cells [32,58]. The central nervous system, especially the motor cortex region, should maintain or increase incentives for related motor neurons to deliver sustained and stable force production [66]. In driving task, the body parts should be coordinated to gain a favorable performance. This coordination leads to increased activity of motor units and the frequency of stimulation. Our results showed significant activation in LPFC, RMC and ROC when driving. Increased brain activation and redistribution of brain resources (i.e., CBF) are partially controlled by neurovascular coupling. The distribution of brain resources can change the activation intensity in each brain area, and the connectivity between brain areas varies accordingly [67]. The decline of synchronization shown in WPCO in interval IV indicated reduced strength of FC in neurological activity among cortex regions.

The WPCO values of LMC-LOC and LOC-RMC in interval V showed significantly lower level in straight driving. The oscillations in frequency interval V correspond to NO-related endothelial metabolic activity [58,68]. NO is a highly important vasoactive substance [69]. The endothelial cell is a basic component of the blood vessel wall. The NO produced and released by endothelial cells and relaxing smooth muscle cells is associated with vasodilatation and vasoconstriction [70]. The local cerebral metabolism is tightly coupled to local brain perfusion, and this coupling is a product of the anatomical and metabolic relationship among neurons, glial cells, and cortical penetrating arterioles that together comprise the neurovascular unit [71]. In short, the NO-related endothelial metabolic activity is tightly integrated with neurogenic activity. This can be shown from the consistent significant increase in WA values of LPFC, RMC and ROC in interval IV and V. The lower WPCO values in interval V indicated a reduced phase-synchronized endothelial activity in LMC-LOC and LOC-RMC.

In addition, the oscillations in the frequency interval VI correspond to NO-independent (probably prostaglandin-dependent) endothelial activity [58]. Different prostaglandins exert different effects on vascular smooth muscle cells; these prostaglandins cause the smooth muscles to regulate blood flow and blood pressure [72,73]. The NO-independent endothelial activity is closely related to neurogenic activity, as shown in significantly increased WA values of LPFC, RMC and ROC in interval IV and VI. Our results show that driving task may cause the phase asynchrony of the NO-independent endothelial activity in the RMC-LPFC, RMC-LMC, RMC-LOC and RMC-RPFC. This asynchrony indicates a decline in FC.

Interestingly, the WPCO values of LPFC-LMC and RPFC-LMC were significantly higher in interval I in straight driving than in the resting state. The heart pumping effect was reflected in the blood oscillation in interval I. The higher level in this interval indicates the enhanced heart output, and the coordination regulation between LPFC-LMC and RPFC-LMC showed an enhancement due to the driving task.

### GC and FC in distracted driving

In contrast to straight driving, the WA values in the states with secondary auditory task declined in all cortex regions except for LPFC in intervals III and V. This interplay between anterior and posterior brain regions is possibly related to a competition for limited resources and attentional reallocation between the anterior, executive attention in multi-tasking and the posterior, visual response attention system [65]. [9] reported that the distracted brain sacrifices important visual attention and alertness functions from the posterior cortex to recruit sufficient brain resources to perform secondary cognitive tasks, which was consistent with our results. The auditory calculation task added to driving raised the GC level in interval II and FC level intervals V and VI between MC and other cortex areas. This change may result from the brain resource reallocation between MC and other brain regions. The reduced parietal lobe activation in the dual task condition reflected a decrease in the spatial computations associated with driving as well as a decrease in spatial attention [16,74]. Increased phase synchronization suggests an enhanced coordinated regulation of respiratory activity, NO-related endothelial metabolic activity, and NO-independent endothelial activity to cerebral circulation between PFC and MC. Besides, the more unstable driving performance was found in Tasks 2 than in the Task 1. The results indicate that the additional auditory task makes mental resources away from driving and leads to deterioration of driving performance.

The additional visual vigilance task induced a loss of cerebral GC in interval I between PFC and MC, complete loss of FC in intervals IV to VI, and declined activation in LPFC and RPFC in interval VI. This finding was different from the effect of the secondary auditory task on the driving task. Some studies demonstrated that brain activities and connectivity vary under different multiple tasks [75–77]. The main determinants may depend on how the two tasks are automated and whether they are on non-overlapping cortical regions [16].

Task 3 induced significantly lower level in GC including in the LPFC-LMC and RPFC-LMC in interval I compared to that in Task 1. Additional visual vigilance task reduced the coordinated regulation of cardiac activity and cerebral circulation between PFC and MC, and may affect substrate delivery and the removal of by-products of metabolism. Similar results were noted in previous research that focused on vigilance task-related changes on brain FC between PFC and sensorimotor areas [42]. The task might affect the functional hyperemia mechanism and further influence driving performance. Besides, the connectivity map indicates significant decline in the PFC-MC, PFC-OC, and MC-OC in interval IV. The long-range connectivity possesses greater context sensitivity and was crucial for task-dependent functions [78]. These synchronism drops reflect the decreased coordination of sympathetic nervous system function in these cortex pairs. In interval V and VI, few FCs existed in the LPFC-RPFC, LMC-RMC, and LOC-ROC. The decreased FC may suggest that the additional visual task induced a desynchronized endothelial regulation activity and affected the neurovascular coupling level between these cortex regions. Moreover, secondary visual tasks seem to have more adverse impact on driving compared with the secondary auditory tasks, as revealed by significantly higher SDS values not just the larger number of driving errors.

Table 2 shows an interesting result. Although the SDS and the DE were on upward trend in distracted driving, their changes were inversely related to changes in WPCO. The △SDS was negatively correlated with the △WPCO, which may imply that the change of FC reduce the changes of SDS in distracted driving state and make speed more stable. The △DE was positively related to the △WPCO and this indicated that the changes of FC have the same trend as the changes of DE. As SDS was a measure of longitudinal driving stability and DE was more focused on the measurement of lateral driving stability [79], the results may indicated that the driver's brain sacrificed the ability of lateral driving adjustment to ensure longitudinal driving adjustment in distracted driving.

In conclusion, the present study provides new insights into the relationship between distracted driving behavior and brain activity. The results may be used for the evaluation of driving ability and assessment of new interfaces and technologies in vehicles.

### Method consideration

The different driving experiments of subjects could have different impact on FC. Studies demonstrated that long-term behavior and recent experience would change the resting-state of FC and might be directly linked to dynamic changes in FC [80,81]. However, the resting-state of FC of drivers with few years of driving experience is similar to the non-drivers [81]. Thus, in order to avoid the interference of this differentiation, subjects who have driver's license but have less than one year of driving experience were recruited. Moreover, the subjects were given 10 minutes to adapt to the simulator before the experiment.

Within the long time window, the functional connectivity exhibit dynamic changes at different time scales. In this study, in order to reveal the phase coherence value of the low-frequency component (VI, 0.005–0.0095 Hz), the task period was set to 20 minutes. In one time series of fNIRS signal, the number of cycles of the high frequency component is larger than that of the low frequency component. The phase difference of the low-frequency component tends to remain constant compared to the high-frequency component, that is, the phase coherence value of the low-frequency component tends to increase [34]. So at least five or more low-frequency cycles should be included in an fNIRS signal to ensure the effectiveness of the WPCO [82]. The minimum frequency is 0.005 Hz, and then the maximum frequency period is 200s. Thus, the minimum measurement time is 1000s. Averaging WA and WPCO in the time domain can improve the accuracy of the low frequency component.

A multichannel fNIRS study illustrated that the signal-to-noise ratio of [HbO_2_] signal was higher than that of [HHb], and it was more pronounced in cognitive tasks [83]. The [HbO_2_] variable appeared to be sufficiently sensitive for representation of attention reduction in the cortical region, whereas the combination of these two variables only improves slight performance in some cases [84]. Therefore, only Delta [HbO_2_] signal was used for WPCO analysis in this study. Since [dHb] signal may be less contaminated by global processes compared with [HbO_2_] [33,85], future studies will consider changes both in [HbO_2_] and [dHb] for better physiological interpretation of the results of FC.

One limitation of the study is the small sample size. The subjects performed the resting-state and tasks in the same order, however, counterbalance experimental design may make the results more scientific. Since the objective of this study is to provide a method for the evaluation of functional connectivity (FC) in relevant cortex areas during driving with distraction, future studies will add more subjects, focus on balanceable procedure and further validate the results. Another limitation is that we did not consider the interference of Mayer waves (0.08–0.1 Hz). Mayer waves are oscillations of arterial pressure occurring spontaneously in conscious subjects at a frequency lower than that of respiration and tightly coupled with the synchronized oscillations of the efferent sympathetic nervous activity [86]. On the one hand, the cerebral oscillations in interval III (0.052 to 0.145 Hz) were suggested to originate locally from the intrinsic myogenic activity of smooth muscle cells in resistance vessels and this myogenic mechanism may be partly under autonomic control [29]. On the other hand, the frequency band III might be contaminated by Mayer wave. In this study, the driving task-related changes in connectivity in interval III mainly reflects the synchronization of neurovascular coupling, that is, the difference of myogenic activity in different cortex areas and the lower synchronization in straight driving compared to resting state. The interference of Mayer waves will be taken into account in future study.

## Supporting information

S1 File This is the data of driving performance in Task 1, Task 2 and Task 3, including SDS (km/h) and number of DE. (XLSX)

S2 File This is the WA data of six frequency intervals of four states. (XLSX)

S3 File This is the WPCO data of six frequency intervals of four states. (XLSX)

S4 File This is the WA data before being integrated in six frequency intervals of four states. The frequency value and the corresponding WA value are stored at the end of the struct.
(MAT)

S5 File This is the WPCO data before being integrated in six frequency intervals of four states. The frequency value and the corresponding WPCO value are stored at the end of the struct.
(MAT)
